# Study protocol of the eating and nutritional habits in athletes and controls longitudinal examination – ENHANCE Study

**DOI:** 10.1007/s44192-026-00544-y

**Published:** 2026-07-30

**Authors:** Anna J. Esser-Seraphin, Vanessa T.M. Nguyen, Sheila Geiger, Georgios Paslakis, Adam Schweda, Hannah Dinse, Eileen Reinemann, Thomas Muehlbauer, Jochen Seitz, Gertraud Gradl-Dietsch, Eva-Maria Skoda, Martin Teufel, Alexander Bäuerle

**Affiliations:** 1 Clinic for Psychosomatic Medicine and Psychotherapy, LVR-University Hospital Essen, University of Duisburg-Essen, Virchowstr. 174, 45147 Essen, Germany; 2 Centre for Translational Neuro- and Behavioural Sciences (C-TNBS), University of Duisburg-Essen, Essen, Germany; 3 Department of Psychosomatic Medicine and Psychotherapy, LWL-University Hospital, Ruhr-University Bochum, Bochum, Germany; 4 Division of Movement and Training Sciences, Biomechanics of Sport, University of Duisburg-Essen, Essen, Germany; 5 Clinic for Child and Adolescent Psychiatry, Psychosomatic Medicine and Psychotherapy, LVR-University Hospital Essen, University of Duisburg-Essen, Essen, Germany; 6 Department of Child and Adolescent Psychiatry, Psychiatric University Hospitals, University of Basel, Basel, Switzerland

**Keywords:** Disordered eating, Eating Disorders, Clinical interviews, Elite sports, Sport-specific risk factors, Sensitivity, Relative Energy Deficit in Sport

## Abstract

**Background:**

Training and body composition requirements in elite sports may elevate eating disorder (ED) risk. Current estimates suggest a range of 1–28% prevalence of ED in elite athletes, reflecting methodological heterogeneity, underreporting, and limited research focus. EDs adversely affect physical health (e.g., osteoporosis, fatigue, injury), psychological well-being, and athletic performance, often persisting beyond athletic careers. Sport-specific demands may obscure ED symptoms, making underdiagnoses likely. This longitudinal study’s objective is to furnish proof for the theoretical model of disordered eating in elite athletes by examining the role of sport-specific indicators, to improve early identification and psychological diagnostics.

**Methods:**

Three-hundred elite athletes from weight-sensitive (ballet, bodybuilding) and less weight-sensitive (soccer, racket sports, basketball) sports will complete assessments at four time points. A subgroup of 90 athletes will participate in clinical interviews. Logistic and hierarchical regressions will identify ED risk indicators and estimate the prevalence of EDs in athletes. Receiver Operating Characteristic (ROC) analyses will assess diagnostic accuracy of single and combined indicators with reporting of sensitivity, specificity, and the Area Under the Curve (AUC). Group differences and measurement invariance of ED instruments will be tested between the athlete group and 300 non-athlete controls.

**Discussion:**

Identifying reliable ED indicators in elite sports may support early intervention. This theory-based approach aims to enhance diagnostic accuracy and athletes’ care.

**Trial registration:**

Prospective registration of the study in the German Clinical Trials Register (DRKS00035100) on 03 February 2025.

**Supplementary Information:**

The online version contains supplementary material available at 10.1007/s44192-026-00544-y.

## Background

Psychological distress in elite athletes gains increased attention in both media coverage and scientific debate [1]. New screening instruments such as the Sport Mental Health Assessment Tool [2] were developed by the International Olympic Committee to better identify athletes at risk for mental health symptoms and allow timely support for athletes [3, 4].

Since eating disorders (EDs) in athletes have a negative impact on health, for example by leading to osteoporosis, higher injury rates, amenorrhea, fatigue, and performance decline [5–8], research on the psychopathology of EDs seems inevitable. The onset of EDs mostly occurs in puberty, which endangers a proper physical development [9]. Affected athletes show high comorbidities with affective-, anxiety- and substance use disorders [10]. ED symptoms often become chronic, leading to health restrictions, direct (e.g. hospitalization, medication) and indirect (e.g. societal production deficit due to premature death, symptom-driven food expenses) health economic costs and a reduced quality of life, even beyond the end of an active career [3, 11]. Early identification is crucial to prevent both performance loss and symptom chronicity.

EDs are reported in 1% to 28% of elite athletes [3], a variability that likely reflects methodological heterogeneity, underreporting, and limited research focus [12]. In addition, elite athletes may express disordered eating (DE) through differential phenotypes, with DE defined as maladaptive eating behaviors and cognitions that do not meet diagnostic criteria for an ED in terms of frequency, duration, or clinical severity [13–15]. Those DE patterns might be influenced by training status, sport discipline, or energy availability. These complexities underscore the need for a multidimensional approach to identify affected athletes [16]. A theoretical model for the psychopathology of EDs in elite sports was developed based on existing knowledge and evidence [16]. It expects the indicators *weight-sensitivity* of the sport-discipline, *weight and shape pressure*, *internalisation of a sports discipline-specific body ideal*, *unhealthy training behaviours and intentions*, and *fatigue* to potentially enhance the probability to have an ED as an athlete [16].

The model considers that athletic success and health risks often coexist in elite sports [17]. Elite athletes face the challenges of adhering to sport-specific eating, and nutritional and training habits to create optimal physical conditions for their high training requirements [16, 17]. Particularly in weight-sensitive sports disciplines (e.g., bodybuilding, ballet dance), the sport context dictates specific body images or weight goals that pressure elite athletes to follow certain dietary and training practices for their health and athletic success [17, 18]. Research in non-athletes on muscularity-oriented behaviours has the potential to improve diagnostic practices in men [19]. These insights may also aid at identifying DE in both men and women athletes, as elite sports place emphasis not only on thinness but also on the targeted modification of body composition, muscularity types, and specific body areas, regardless of sex [20].

In some sports disciplines, periods of *Adaptable Low Energy Availability* (adaptable LEA) or *Functional Overreaching* are used to enhance athletic performance, whereby athletes modulate their body weight or challenge their athletic limits before restoring sufficient energy availability or recovery [4]. Hunger does not reliably reflect the elevated energy and nutritional needs of physically active individuals, making athletes prone to inadvertent energy deficits [21], especially when nutrition knowledge is not sufficient [22–24]. In addition, the reinforcement athletes receive within the sport context for internalising body images by aligning their eating and training habits, may promote DE [25–27] and other health-compromising practices such as obsessive healthy eating [28], or the use of anabolic steroids [29]. If training load is not compensated with nutritional energy or regeneration, then mental, endocrine, and metabolic health impairments can occur [30, 31], described as *Relative Energy Deficit in Sport* (RED-S) or *Overtraining Syndrome* (OTS). Each syndrome has traditionally focused on either nutritional or recovery-related aspects; however, recent research has demonstrated substantial overlap in their etiology, symptom presentation, and diagnostic characteristics [32]. Previous approaches investigated potential physical markers for RED-S or OTS [33, 34], e.g., increased visceral fat in combination with hormone imbalances [35]. Diagnosis of these syndromes remains challenging due to heterogeneous symptom presentations [36, 37]. Fatigue is a key symptom, often accompanied by reduced performance and psychological strain [21, 26, 38, 39].

As the consequences of eating and training regimes can be both protective and damaging [31], DE may remain undetected or misinterpreted as sport-typical behaviour [16]. Conventional diagnostic criteria might especially be ineligible to diagnose EDs in elite athletes, as those are not tailored to sport-specific requirements [40]. While the above-mentioned model of Esser-Seraphin and colleagues has yet to be empirically tested, it may function to fill this research gap by providing a promising, evidence-based approach for the use in sport-specific healthcare and clinical practice.

### Objectives

The *primary objective* of the study is to evaluate the validity of the model for pathological eating in elite sport [16]. The indicators (weight-sensitivity, weight- and shape pressure, internalisation of a sports discipline-specific body ideal, unhealthy training intentions and behaviour, fatigue) specified in the model are expected to explain variance in ED symptomatology (measured by EAT-26) and/or increase the likelihood of an ED (clinical interviews) in elite athletes. The *second aim* is to investigate whether the diagnostic accuracy of ED identification in elite sports can be improved by combining standardized instruments (e.g., EAT-26) with sport-specific indicators (e.g., ART). The goal is to empirically derive cut-off scores that prioritize high sensitivity to minimize the risk of false negatives, while maintaining acceptable levels of specificity. The *third aim* is to estimate the prevalence of EDs in elite athletes by both the number of diagnosed cases in this cohort and ED instruments. The prevalence in weight-sensitive sports disciplines is hypothesised to be higher compared to less weight-sensitive sport-disciplines and compared to a control group. *Further study objectives* are the identification of protective factors (e.g., sport type, nutrition knowledge) for DE, the investigation of the predictive validity of the indicators, and the association of physiological markers (e.g., body fat percentage) with DE in elite sports. Methodologically, this study aims at investigating whether conventional instruments measure ED symptoms differently in elite athletes than in the control group.

## Materials and methods

To the best of our knowledge, there is no specific guideline available for reporting study protocols of longitudinal diagnostic accuracy studies [41]. Given this is a non-interventional, diagnostic accuracy study, the “Standards for Reporting of Diagnostic Accuracy Studies” [42] guidelines served as the best available alternative to prepare this study protocol. Prior to recruitment (03 February 2025), the study had been registered at the German Clinical Trials Register (DRKS00035100). If important changes are made to the study protocol, the ethics committee will be informed, and the registration of the study will be updated.

### Study design and procedure

**Fig. 1:**
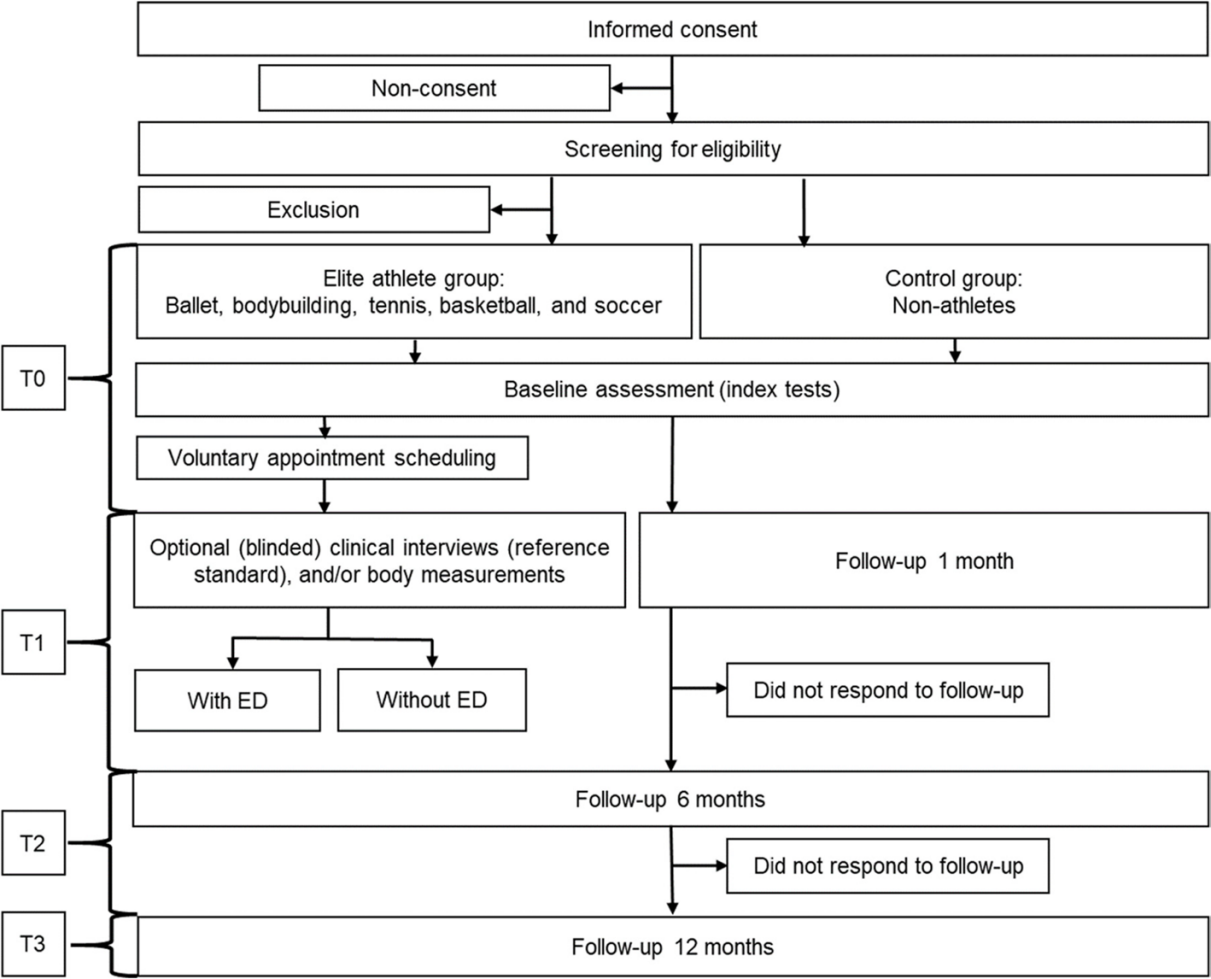
Flow chart of the study. Notes: T0: Baseline assessment, T1-T3: Time points for follow-up assessments, ED: Eating disorder, blinded: Regarding the scores of the index tests

ENHANCE (Eating and Nutritional Habits in Athletes and Controls — a longitudinal Examination) is a prospective, non-interventional, longitudinal study combining quantitative assessments and semi-structured clinical interviews to evaluate the validity of our previously published theoretical model [16] and assess the prevalence of EDs among elite athletes, including a non-athlete control group for comparative analysis. At four assessment points (T0 – T3, see Fig. 1), sociodemographic, health, training, nutritional, and psychological data will be collected via digital assessments (created with Unipark software, Tivian XI GmbH) as self-reports from both the athlete and non-athlete group. Additionally, a subgroup of athletes will be invited to participate in clinical interviews and body measurements.

After providing electronic informed consent, participants can access an eligibility screening by following the study link. Based on the eligibility screening, the participants are assigned to either the elite athletes or the control group, or will be excluded from the study (see section participant eligibility and recruitment). After completing the baseline assessment, controls can exit the survey, while elite athletes will be forwarded to an appointment booking system. There, they can optionally schedule an appointment for a clinical interview (online or in person, in German or English) and/or the body measurements approximately one month after baseline assessment. Trained specialists will conduct the clinical interviews and body measurements. Body measurements (i.e., body fat percentage) can also be conducted by an external health care professional chosen by the participant. Interview and body data are documented in an electronic case report file. Links to access the follow-up assessments are send via email by the study staff. All emails and reminders are standardized. Data of each assessment point will be linked using pseudonymized participant codes.

### Participant eligibility and recruitment

All participants are required to speak and understand English fluently to be included in the study. This study is aimed at elite athletes who (a) practice either ballet, bodybuilding, racket sports, soccer, or basketball, (b) organise their daily life around their sport and (c) regularly participate in competitions or performances [1, 13, 36, 43, 44]. To be assigned to the elite athlete group, the training must have been practiced for at least six months with more than 300 min/week at moderate or higher intensity [36]. Participants, who fulfil the criteria for classification as elite athletes and practice one of the sports disciplines mentioned above, are included in the elite athletes group. Participants who do not meet the criteria of the elite athlete group are assigned to the control group. Participants in the control group may also train regularly but should not match the elite athletes’ criteria. If the participants fulfil the criteria to be considered elite athletes, but do not practice any of the sports disciplines examined in this study, they will be excluded from the study.

Participants are recruited purposefully at sports facilities, sports boarding-houses, ballet academies, ballet companies, sports clubs, fitness fairs, sports events, and on social media. Among other recruitment strategies, we search Instagram accounts affiliated with ball sport-, ballet-, or bodybuilding associations like the International Federation of Bodybuilding and Fitness. Moreover, ballet companies or academies are contacted to inform potential eligible participants about the study and distribute the recruitment material.

A screening prior to baseline assessment enrols eligible participants. They will be asked to provide their contact details in a survey link separate from study data, which is used to contact participants one, six and twelve months after baseline assessment for follow-up assessments (see Table 1.). Participation in the clinical interviews is voluntary for the athlete group.

**Table 1:** Assessment Schedule

				Planned assessment time points								
				Athletes					Controls			
Criteria				T0	T1	T2	T3		T0	T1	T2	T3
	*Eating disorder (A) and Disordered eating (B)*											
			Clinical interviews		X							
			Eating Attitudes Test-26	X		X	X		X		X	X
			Eating Disorder Examination Questionnaire		X					X		
			Brief Eating Disorder in Athletes Questionnaire	X	X	X	X		X	X	X	X
Indicators				T0	T1	T2	T3		T0	T1	T2	T3
		*Weight and shape pressure (E)*										
			Weight Pressure in Sport in Males/ Females	X			X					
		*Body image (F)*										
			Muscle Dysmorphic Inventory	X			X		X			X
			Subscale Drive for Thinness Eating Disorder Inventory 3	X			X		X			X
		*Training habits (G)*										
			Athletes’ Relationships with Training Scale	X		X	X					
			Rating of Perceived Exertion	X	X	X	X		X	X	X	X
		*Fatigue and mood disturbances (H)*										
			Profile of Mood Scale	X			X		X			X
			Rating of Fatigue	X	X	X	X		X	X	X	X
			Endocrine and Metabolic Responses on Overtraining Syndrome Tool	X								
			Low Energy Availability in Females/ Males	X		X*	X*		X*		X*	X*
Other measurements				T0	1T	T2	T3		T0	T1	T2	T3
		*Nutritional knowledge and obsessive healthy eating (I)*										
			Abridged Nutrition for Sport Knowledge Questionnaire		X					X*		
			ORTO-Revised version		X					X		
		*Psychological strain (J)*										
			Athlete Psychological Strain Questionnaire	X	X	X	X					
			Patient Health Questionnaire 4	X	X	X	X		X	X	X	X
		*Body measurements (K)*										
			Bioimpedance analysis, blood pressure, heart rate, oxygen saturation	X		X	X					

Notes: TO: Baseline assessment, T1-T3: Follow-up assessments. *ORTO*: Instrument measuring orthorexic thoughts and behaviours. X *: Only assessments of selected subscales. Controls only fill in non-sports-specific items. Body measurements: Body mass index, heart rate, blood pressure, oxygen saturation, waist-hip ratio and body composition. Letters in brackets: In reference to the order in section 2.3. Outcome measures

### Outcome measures

The primary outcome is the additional variance explanation in the psychopathology of eating behaviour in the elite sports group by the indicators (∆R²) of our theoretical model of DE [16]. The criteria of the regression models will be measured (A) binary conducting clinical interviews (ED present or absent) and (B) continuous using an ED instrument (severity of ED symptomatology).

*A)* Clinical interviews serve as a reference test. Trained specialists will assess participants for EDs (anorexia nervosa, bulimia nervosa, binge-eating disorder, and others) using ICD-11 criteria [14] in a ~ 30-minute interview. Based on the indicator internalization of a sport-specific body ideal, including muscular body images that can potentially contribute to EDs in elite sports, the criteria for muscle dysmorphia proposed by Pope et al. [45] are also part of the interview. ICD-11 criteria will be evaluated using selected questions of the ED examination interview from Lichtenstein et al. [26], allowing a sports-specific exploration of the fear of weight gain or behaviours to prevent weight gain, weight and shape concerns, food-related loss of control, exercise behaviour, and compensatory behaviours. Additionally, performance enhancing substance use, obsessive healthy eating, and physiological consequences of low energy availability in elite sports like reduced sex drive or menstrual dysfunction are evaluated [26, 46, 47]. In addition to the identification of EDs associated with clinically relevant psychosocial and physical impairments, borderline cases will be documented as DE presentations [48]. In comparison to the ICD-10, the ICD-11 might facilitate diagnosis in men by removing the endocrine disorder criterion [14]. Neither the interviewers nor the participants are informed about the results of the index tests.

*B)* The Eating Attitudes Test 26 [EAT-26; [49] evaluates the frequency of DE behaviours on a 6-point-scale with a cut-off of 12.66 indicating clinical risk in athletes [50, 51]. The EAT-26 is administered at T0, T2 and T3 and is used as a measure of ED symptom severity in the hierarchical regression analyses.

Self-reported DE will also be assessed by the Eating Disorder Examination Questionnaire [EDE-Q, [52] in both groups and by an additional third instrument only in the elite athlete group. The EDE-Q is a valid and reliable EDs instrument with 28 items and four subscales (restraint, eating concern, weight concern, shape concern). Participants indicate the frequency of eating behaviours over the past 28 days. In a Danish elite athlete cohort, the EDE-Q showed 90% sensitivity and 100% specificity with a cut-off of 2.3 [26]. The Brief Eating Disorder in Athletes-Questionnaire [BEDA-Q; [40] is validated for women athletes and consists of nine items indicating DE with a cut-off of 4 [40]. The first six items are adopted from the Eating Disorder Inventory 2 EDI-2 [53] and can be rated on a 6-point-scale. The three last items capture dieting regimes and frequency. The area under the curve (AUC) was 0.73 (98% CI, 0.52–0.93). Overlapping items of the instruments (e.g., Item 11 in the EAT-26 is the item 2 in the BEDA-Q and item 32 of the EDI-3) are only assessed once per survey. Both the EDE-Q and the BEDA-Q are used to assess the convergent validity of the EAT-26. They are not included as model criteria due to differences in assessment time points and item overlap with other instruments operationalising constructs in the theoretical model.

The regression models’ predictors consist of (C) sociodemographic-, health-, and sport-specific data, (D) the weight-sensitivity of the sports discipline, (E) the athletes’ perception of weight and shape pressure, (F) the internalisation of sport-specific body ideals (drive for thinness and muscularity striving behaviour), (G) training load, intentions and behaviours, and (H) the experience of fatigue and mood disturbances.

*C)* Sociodemographic data comprise sex, gender, age, nationality, occupation, and financial situation. Health data includes previous counselling or treatments and previous ED diagnoses. As the study targets elite athletes, the surveys include self-developed sport-specific items for elite athletes, such as sports discipline, current intention to modulate body weight, training phase and amount in their sports discipline. Controls are asked to report general training frequency and duration. Both groups indicate nutritional and movement data in general.

*D)* The classification of weight-sensitivity into weight-sensitive (ballet) and less weight-sensitive (ball games) sports are adopted from Martinsen and Sundgot-Borgen [12]. The authors do not classify bodybuilding, but in this study it is considered a weight-sensitive sport, as the evaluation of physical appearance is regarded as the athletes’ performance [54, 55].

*E)* The Weight pressure in Sport Scale for men athletes [WPS-M; [56] and for women athletes [WPS-F; [57] are instruments that assess pressures from coaches and peers and include subscales specific to sex with 16 items for women and 14 items for men rated on a 6-point scale. In this study, items with the term “team members” is changed to “sport colleagues”, as most of the sports disciplines included in this study are individual sports.

*F)* Internalisation of body ideals is assessed by two validated instruments. The Muscle Dysmorphic Disorder Inventory (MDDI) measures the internalisation of a muscular body ideal [MDDI; [58] and includes 13 items across three subscales rated on a scale from 1 (never) to 5 (always). Based on the suggestion of Compte et al. [59], item five (“I think my chest is too small”) is adjusted to “I think my chest (muscle) is too small” to avoid the reference to breast volume [59]. The MDDI was used in an elite athlete cohort with men and women, showing good internal consistency (Cronbach’s α = 0.824), and categorizing individuals with scores ≥ 39 at risk for muscle dysmorphia [60, 61]. The internalisation of a thin body ideal is measured by the subscale of the Eating Disorder Inventory 3 Drive for Thinness [DT; [62, 63], which consists of 7 items rated on a scale from 0 (= never) to 5 (= always). Scores > 14 are considered as high DT [64]. EDI-3 items for weight history are used.

*G)* Training intentions and behaviour among athletes are measured with the Athletes’ Relationships with Training Scale [ART; [65] consisting of five subscales. Items 1–9 are rated on a scale from 1 (never) to 5 (always) and items 10–15 are rated on a scale from 1 (strongly agree) to 5 (strongly disagree). Scores ≥ 48 are considered as attitudes indicating DE. Additionally, training intensity on average over the last week is measured by a modified instruction for the Rating of Perceived Exertion [RPE; [66–70]. This single item scale assesses subjective physical effort on a scale from 1 (light activity) to 10 (maximal effort activity). Classification into easy (RPE ≤ 3), moderate (RPE 4–6), and hard (RPE ≥ 7) training intensity followed the suggestions of Ieno et al. [71].

*H*) Experience of fatigue as symptom of RED-S is measured by modified versions of the Low Energy Availability in Males Questionnaire [LEAM-Q; [46] and its counterpart for women, the Low Energy Availability in Females Questionnaire [LEAF-Q; [47]. Both instruments are self-report measures designed to detect physiological symptoms indicative of LEA in athletes. In the present study, selected sections.  (1, 2, 4, and 5) from the full LEAM-Q are administered to both men and women athletes. LEAF-Q subscales are included to collect use of contraceptives, and menstrual history and dysfunction in women athletes [47]. The pictorial rating of fatigue scale [ROF; [72] measures fatigue with response rates from 0 (not fatigued at all) to 10 (total fatigue & exhaustion- nothing left). The ROF scale can be used in the sports context to monitor athletes’ regeneration and ability to perform. Fatigue as symptom of Overtraining is assessed with the Endocrine and Metabolic Responses on Overtraining Syndrome clinical tool [EROS; [36]. The instrument consists of nine parameters [3 items on nutritional information, 4 Profile of mood states items, 2 items on performance impairment, and 1 item on sleep quality; 74], which adds up to a score that rules out OTS (0–2), remains inconclusive (3–5), or diagnoses OTS (6–9). Overlapping items with other scales are only assessed once. Mood disturbances are assessed by a short form of the profile of mood states [POMS; [73]. The questionnaire consists of 40 items with subscales rated on a scale from 0 (not at all) to 4 (extremely). The POMS was used in elite athlete cohorts to evaluate psychological distress, subjective training response, and exhaustion [74].

Except for the regression model’s predictors and criteria, *secondary outcomes* like (I) nutritional knowledge, obsessive healthy eating, sport-specific eating motives, and (J) psychological strain are assessed. Moreover, the elite athletes can participate in (G) body measurements.

*I)* The Athlete Nutrition Sport knowledge Questionnaire [ANSQK; [75, 76] measures general and sports nutrition knowledge with 37 items [75]. Overall, the ANSKQ is considered a reliable and valid measurement in which participants evaluate their weight management, macronutrients, micronutrients, supplementation, sport nutrition, and alcohol [75]. The ORTO-R [75, 76] is a revised version of the ORTO-15 to measure orthorexic thoughts and behaviours with six items rated on a four-point-scale [77–80]. The ORTO-R has been used in a sample of competing athletes showing measurement invariance in comparison with participants less active [81]. Sport-specific eating and nutritional behaviour is assessed with self-developed items.

*J)* The Athlete Psychological Strain Questionnaire (APSQ) measures psychological strain in elite athletes [82] with ten items [83] rated on a scale from 1 (none of the time) and 5 (all of the time). APSQ scores of ≥ 20 are considered as ‘very high’ psychological strain [83]. The Patient Health Questionnaire (PHQ) is a validated 4-item instrument, which measures severity of depressive symptomatology and anxiety with PHQ-2 ≥ 3 as risk for depression and GAD-2 ≥ 3 as risk for anxiety [84, 85].

*K)* The body measurements include the BMI (kg/m²), body composition, waist-hip ratio, blood pressure, and heart rate. The BMI assesses the nutritional status in adults and serves as a diagnostic criterion for EDs according to ICD-11 [86]. In adults, a BMI between 18.5 and 24.9 kg/m^2^ is considered as normal weight [87]. If participants do not undergo the body measurements, the BMI will be calculated from a self-report of the participant’s current body height and weight. A Seca mBCA 515 scale (Bioimpedance analysis) in the Clinic for Psychosomatic Medicine and Psychotherapy, LVR-University Hospital Essen (Germany) is used to measure body composition including fat-free mass, skeletal muscular mass, total body water, extracellular water, visceral fat, and body fat percentage adjusted for age, ethnicity, and gender to consider nutritional behaviour and needs over time [88], and physiological indicators of OTS symptoms [35]. The Seca scale uses Physical activity levels (PAL) to calculate total energy expenditure. As athletes move more than non-athletes, we derive PAL values from the participant’s basal energy expenditure and the Metabolic Equivalent of Task values for minutes of selected activities executed per day averaged over a week [89]. Systolic and diastolic blood pressure is measured with a digital blood pressure monitor (model name: Boso Medicus X Blood Pressure Monitor with All Basic Functions) after ten minutes of sitting. The oxygen saturation and heart rate (beats per minute) will be assessed with a pulse oximeter (from UEBE Medical GmbH, model: visomat PO_50_) at rest. Scales and devices from external health care professionals may vary, which will be documented.

### Sample size calculation

Sample size planning is based on the statistical verification of the primary objective. To estimate the required sample size, we conducted an a-priori power analysis using G*Power 3.1.9.7 [90]. We expect weight pressure, internalisation of a sport-specific body image, unhealthy training attitudes and behaviours, and physical and psychological fatigue symptoms to predict DE in athletes [16]. To assess the validity of the model, a hierarchical regression model will be computed for men and women respectively. Thus, the number of predictors tested will be six, and total predictors of the regression model include nine predictors with sociodemographic data. Effect sizes for men athletes were found to be smaller than for women athletes [91]. However, the strive for muscularity is not considered in most studies investigating DE in athletes, which has been described as a promising indicator to explain variance in ED symptomatology especially in men [92]. Consequently, effect size for men and women athletes are expected to be medium (Cohen’s *f*^2^ = 0.15) in the present study [3, 93]. The power for the sample size calculation was determined to 1-*ß* = 0.95. This results in a sample size of *n* = 146 for men and women athletes for the quantitative data assessment, respectively. Sample size per sports discipline (ballet, bodybuilding, and ball sports) will be balanced with approximately *n* = 100 each (total *n* = 300). For the comparison with the control group, *n* = 300 control participants are also sought.

The minimum sample size for the logistic regression was calculated using Wald’s-z test with fatigue as an exemplary predictor of least interest. The power curves showed the required sample sizes under variation of two parameters (see appendix Fig. 2). The minimum sample size for the logistic regression was balanced with the feasibility to perform clinical interviews with athletes who lack time due to strict training schedules while still reaching an acceptable power. Research findings on the prevalence of EDs in athletes range from 1.00 to 28.00% [3, 93], depending on sex, sport-discipline, weight-sensitivity of the sport, and self-report measures [94]. Rousselet et al. [93] considered that standard criteria are not reliable to diagnose EDs in athletes and revealed a proportion of athletes with an ED of 32.90%, independently of sex [93]. This prevalence is assumed to be realistic because modified ED criteria, such as those used in this study, could also capture the number of unreported cases. Hence, the prevalence of EDs in athletes as the base probability (*P*_0_) was estimated at 30.00%. It is assumed that variance explained by other covariates (*R*^2^ other) adds up to 20.00%. The effect size is estimated as medium odds ratio (OR) = 4.00 and the power is determined at 1-*ß* = 0.80 [95]. This results in a sample size of *n* = 43 for men and women athletes, respectively (*N* = 86). Thus, in bodybuilding, ballet, and ball games, approximately *n* ~ 30 interviews will be conducted, respectively.

### Planned data analysis

Statistical analysis will be performed with updated versions of R and SPSS. *P*-values of less than 0.05 will indicate statistical significance. Effect sizes will be interpreted after Cohen [96]. Variables of interest will be reported with descriptive statistics. Group differences in the sample and psychometric properties of the scales will be assessed using corrected item-total correlations and Cronbach’s α to evaluate internal consistency in the total sample and across subgroups. Conceptually related constructs, particularly fatigue-, recovery-, and energy availability-related measures (see section 2.3 Outcome measures H), will be evaluated for intercorrelations and, where appropriate, combined into standardized composite indices to reduce redundancy and mitigate multicollinearity.

Primary hypotheses will be tested using multivariate hierarchical linear and logistic regression models within a theory-driven blockwise approach, predicting either ED symptoms (continuous) or ED (binary). Given sex-specific validation of some instruments, regression analyses will be conducted separately for men and women athletes. Sociodemographic and sport-related control variables (e.g., age, training duration, and training intensity) will be entered in the first block. Subsequently, theoretically defined predictor blocks will be entered separately to examine their incremental contribution to the prediction of DE. These blocks will include weight sensitivity, weight and shape pressure, internalization of a thin body ideal, muscularity striving behavior, unhealthy training intentions and behaviors, and fatigue-related measures (see section 2.3 Outcome measures C-H). Hierarchical and logistic regression analyses will be conducted using primarily complete-case analysis (listwise deletion). Indeterminate index test or reference standard results will be documented transparently and excluded from primary diagnostic accuracy analyses. To consider suppression effects, multicollinearity will be examined.

Further, Receiver Operating Characteristic (ROC) curves and the AUC will be used to assess the diagnostic accuracy of individual and combined instruments to address the second aim. An AUC of 0.50 indicates no discriminative ability, values between 0.80 and 0.89 reflect good accuracy, and values between 0.90 and 1.00 indicate excellent accuracy. For each instrument or combinations of significant indicators from previous regression models, sensitivity (true positives / [true positives + false negatives]) and specificity (true negatives / [true negatives + false positives]), positive and negative likelihood will be calculated. To determine the optimal cut-off score for each instrument, Youden’s index [97] will be applied, identifying the point on the ROC curve that maximizes the sum of sensitivity and specificity (Youden’s J = Sensitivity + Specificity – 1). In addition, to minimize the risk of false negatives and thus the likelihood of overlooking athletes with clinically relevant eating pathology, alternative cut-off scores will also be evaluated by prioritizing a sensitivity of at least 80%, while maintaining an acceptable specificity threshold (e.g., ≥ 70%).

Significant predictors with the highest diagnostic accuracy will be included in logistic regression models as independent variables and ED diagnosis as dependent variable to calculate ORs. Based on this model, individual probabilities for the presence of an ED will be calculated for each participant in the athlete sample. The estimated prevalence is defined as the mean of these predicted probabilities. Mean differences of the probabilities (dependent variable) will be compared between the subgroups (independent variable) with ANOVAs for aim three. If the requirements for the analysis are not matched, we will conduct adequate alternative tests.

The *methodological examination* will be conducted by performing a measurement invariance using multi-group confirmatory factor analysis (CFA) to evaluate whether the factor structure of the ED instruments is equivalent between athletes and controls. ROC curves will be compared between athletes and controls, to assess if the identification of participants with or without an ED is better in controls than in athletes.

To identify risk or protective factors affecting DE over time as stated in *further aims*, among others linear-mixed models will be conducted. These models assume that data are missing at random (MAR). Attrition bias will be evaluated by comparing baseline characteristics between participants with complete and incomplete follow-up data. If systematic differences are observed, sensitivity analyses will be considered to assess the robustness of the findings.

Exploratory analysis may include interaction testing, pathway analysis (e.g., structural equation modelling), latent class models, or network analysis to examine complex relationships among predictors and ED symptoms. If systematic differences are observed between athletes participating in the clinical interviews and those participating in surveys only, inverse probability weighting may be applied as a sensitivity analysis to account for potential selection bias by weighting individuals according to their estimated probability of interview participation. If substantial overlap between related constructs is detected, sensitivity analyses using alternative operationalisations (e.g., separate versus composite predictors) will be conducted to evaluate model stability and robustness. Sensitivity– and subgroup analyses may additionally examine models combining clinically diagnosed EDs and subclinical DE presentations. Potential sex differences among athletes in the factor structure of ED instruments will be assessed through a measurement invariance analysis.

### Ethics, dissemination, and data protection management

This study has been approved by the Ethics Committee of the University Duisburg-Essen (24-12041-BO). Data collection, storage, and analysis follow the General Data Protection Regulation [98] regulations and the standard operating procedures of the LVR-University Hospital Essen, Germany.

This is a survey-based study supplemented by optional, voluntary clinical interviews and body measurements. No physical or psychological risks are anticipated. Participants receive detailed study information, including data protection, the voluntary nature of the study, and compensation before providing digital informed consent. In case an ED or clinically concerning low body weight are detected, participants will be offered counselling and treatment options through the Clinic for Psychosomatic Medicine and Psychotherapy, LVR-University Hospital Essen, Germany. Study staff is trained in confidentiality and data protection. The study is conducted in accordance with the ethical principles outlined in the Declaration of Helsinki [99]. Pseudonyms link data across assessment points. To protect data while enabling sample size tracking and follow-up assessments, a separate list includes pseudonyms, contact details, participation dates, and group assignments. This list is kept apart from the study data. Results will be shared via peer-reviewed journals, conference presentations, and with relevant stakeholders. Key findings will also be published in lay terms online and promoted through social media.

## Discussion

For the first time, a theoretical model for DE specifically tailored to elite athletes was developed [16]. Building on this theory-based framework, the ENHANCE study aims to empirically examine the model by identifying indicators that can distinguish athletes with and without an ED. This study may not only advance our understanding of EDs in elite sports but may also provide a solid foundation for the development of targeted, evidence-based (preventive) health measures.

The marked inconsistency in prevalence estimates of EDs among elite athletes may be attributable to limited research focus on the topic and to methodological and structural challenges inherent in studies conducted in elite sports [40]. The strengths of the present study address some of these limitations. Its theory-driven approach generates precise hypotheses and specifies relevant indicators for EDs in elite sports. Embedded within a longitudinal design, the theoretical model for ED symptomatology in elite sports supports the interpretation of the results and helps to distinguish between fluctuations and meaningful, theory-based changes [16].

Discrepancies in the results of self-report instruments and clinical interviews [12] suggest underdiagnosis, especially as clinical interviews are rarely applied in elite sports settings [26, 40]. By incorporating clinical interviews as reference tests, this study enhances diagnostic validity and enables more reliable prevalence estimates across weight-sensitive and less weight-sensitive disciplines. The inclusion of a non-athlete control group further allows examination of whether DE manifests differently in athletes.

The limited findings available on EDs in male athletes may be partly due to underdiagnosis likely driven by the use instruments primarily validated in women [20, 40, 51] and a deviation in the expression of ED symptoms [17, 27]. By including a muscular body ideal and using ICD-11 criteria, this study might more reliably diagnose ED in men athletes, helping to overcome stigma and improve support [26, 100, 101].

Many studies recruit athletes from various sports disciplines and generalise their results across all elite sports [102], instead of considering sports discipline-specific demands [103]. The focus on distinct sports disciplines permits nuanced analysis of differences in ED psychopathology among those disciplines, and particularly in relation to weight sensitivity, while at the same time reducing generalisability across the broader spectrum of elite sports.

However, methodological challenges remain. Research in elite sports is constrained by a highly selective group with limited availability due to demanding training schedules. This often results in small sample sizes, which reduces statistical power and hinders the detection of small but potentially meaningful effects [104, 105]. To maximize sample size and utilize validated instruments, the online assessments are conducted in English. However, including non-native English speakers may introduce bias due to potential comprehension difficulties and reduce generalisability. To monitor this, participants are asked to report their nationality. Additionally, participant drop-out across the four measurement points is a concern; to mitigate this, reminder notifications will be sent and an expense allowance will be provided. Framing eating behaviour as the primary research focus and the employed recruitment strategies may introduce self-selection bias, attracting athletes who are either currently experiencing DE or are entirely unaffected, thereby limiting the sample’s representativeness. As participants must be informed about the study’s content, this limitation cannot be fully avoided. To mitigate bias, recruitment materials deliberately avoided the term ED and instead referred to eating behaviour, thus better reflecting the study’s broader scope beyond clinical DE. Voluntary participation in clinical interviews improves feasibility but may again attract those with greater symptom burden.

This study may hold several potential benefits for both research and applied settings in elite sport. By combining clinical interviews with longitudinal assessments, it aims to improve the diagnostic accuracy of ED identification, addressing current limitations in athlete-specific instruments. These insights may inform early, targeted interventions and contribute to the development of evidence-based guidelines for health professionals working in elite sports, with the potential to enhance athlete care and long-term well-being.

## Supplementary Information

Supplementary Material 1. STARD Checklist

## Figures and Tables

**Fig. 2:**
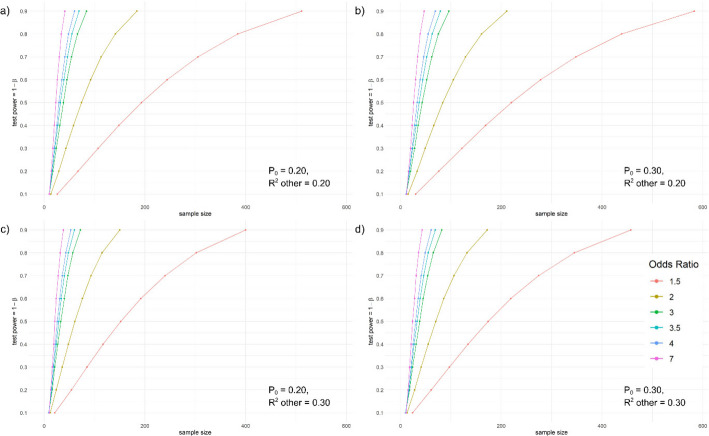
**a** - **d**: Power curves vs. sample size for the logistic regression. *Notes*: Logistic regression with the binary outcome variable (0/1: absence/presence of an ED). *P*_0_: Base probability of having an ED without the influence of the predictors in the model. *R*_2_ other: Explained variance by other covariates. *P*_1_: Probability to have an ED influenced by the predictor. Effect size: Odds ratio (OR). OR < 1.68 - very small, 1.68 < OR < 3.47 – small, 3.47 < OR < 6.71 – medium, OR > 6.71 – large [95], OR = *exp(ß1)* = (*P*_1_/1- *P*_1_)/( *P*_0_/1-*P*_0_). a)-d): Variation of *P*_0_ (either 0.20 or 0.30) and *R*_2_ other [either 0.20 or 0.30; 100]

## Data Availability

No datasets were generated or analysed during the current study.

## Abbreviations

ANOVA: Analysis of variance
ANSKQ: Athlete Nutrition Sport Knowledge Questionnaire
ART: Athletes’ Relationships with Training Scale
AUC: Area under the curve
BEDA–Q: Brief–Eating–Disorder in Athletes Questionnaire
BMI: Body Mass Index
CFA: Confirmatory Factor Analysis
DE: Disordered Eating
DMC: Data Monitoring Committee
DRKS: German Clinical Trials Register
ENHANCE: Eating and Nutritional Habits in Athletes and Controls–a longitudinal Examination
EAT–26: Eating attitudes test–26
ED: Eating Disorder
EDE–Q: Eating Disorder Examination Questionnaire
EDI: 3–Eating Disorder Inventory 3
EDSA: Eating Disorders Screen for Athletes
EROS–Tool: Endocrine and Metabolic Responses on Overtraining Syndrome clinical Tool
FFQ: Food frequency and dieting questions
LEA: Low Energy Availability
LEAF–Q: Low Energy Availability in females Questionnaire
LEAM–Q: Low Energy Availability in males Questionnaire
MDDI: Muscle dysmorphic disorder inventory
MAR: Missing at random
OTS: Overtraining Syndrome
PAL: Physical activity levels
PHQ–4: Patient Health Questionnaire
POMS: Profile of mood states
RED–S: Relative Energy Deficit in Sport
ROF: Rating of Fatigue Scale
ROC: Receiver Operating Characteristics
RPE: Rating of perceived exertion
SOP: Standard Operating Procedure
WPS–F: Weight pressure in sport scale for female athletes
WPS–M: Weight pressure in sport scale for male athletes

## References

[1] Geiger S, Jahre LM, Aufderlandwehr J, Krakowczyk JB, Esser AJ, Mühlbauer T, et al. Mental health symptoms in German elite athletes: a network analysis. Front Psychol. 2023;14. 10.3389/fpsyg.2023.1243804. 10.3389/fpsyg.2023.1243804 PMC10706480 38078219

[2] Gouttebarge V, Bindra A, Blauwet C, Campriani N, Currie A, Engebretsen L et al. International Olympic Committee (IOC) Sport Mental Health Assessment Tool 1 (SMHAT-1) and Sport Mental Health Recognition Tool 1 (SMHRT-1): towards better support of athletes’ mental health. 2021. 10.1136/bjsports-2020-102411 10.1136/bjsports-2020-102411 PMC7788187 32948518

[3] Gouttebarge V, Castaldelli-Maia JM, Gorczynski P, Hainline B, Hitchcock ME, Kerkhoffs GM, et al. Occurrence of mental health symptoms and disorders in current and former elite athletes: a systematic review and meta-analysis. Br J Sports Med. 2019;53:700–6. 10.1136/bjsports-2019-100671. 31097451 10.1136/bjsports-2019-100671 PMC6579497

[4] Mountjoy M, Ackerman KE, Bailey DM, Burke LM, Constantini N, Hackney AC et al. 2023 International Olympic Committee’s (IOC) consensus statement on Relative Energy Deficiency in Sport (REDs). Br J Sports Med. 2023;57:1073–98. 10.1136/bjsports-2023-106994 10.1136/bjsports-2023-106994 37752011

[5] Ackerman KE, Holtzman B, Cooper KM, Flynn EF, Bruinvels G, Tenforde AS, et al. Low energy availability surrogates correlate with health and performance consequences of Relative Energy Deficiency in Sport. Br J Sports Med. 2019;53:628–33. 10.1136/bjsports-2017-098958. 29860237 10.1136/bjsports-2017-098958

[6] Edama M, Inaba H, Hoshino F, Natsui S, Maruyama S, Omori G. The relationship between the female athlete triad and injury rates in collegiate female athletes. PeerJ. 2021;9:e11092. 10.7717/peerj.11092. 33868810 10.7717/peerj.11092 PMC8034341

[7] Holtzman B, Ackerman KE. Measurement, Determinants, and Implications of Energy Intake in Athletes. Nutrients. 2019;11:665. 10.3390/nu11030665. 30893893 10.3390/nu11030665 PMC6472042

[8] Mountjoy M, Sundgot-Borgen J, Burke L, Ackerman KE, Blauwet C, Constantini N, et al. International Olympic Committee (IOC) Consensus Statement on Relative Energy Deficiency in Sport (RED-S): 2018 Update. Int J Sport Nutr Exerc Metab. 2018;28:316–31. 10.1123/ijsnem.2018-0136. 29771168 10.1123/ijsnem.2018-0136

[9] Sundgot-Borgen J. Risk and trigger factors for the development of eating disorders in female elite athletes. Med Sci Sports Exerc. 1994;26:414. 8201895

[10] Åkesdotter C, Kenttä G, Eloranta S, Håkansson A, Franck J. Prevalence and comorbidity of psychiatric disorders among treatment-seeking elite athletes and high-performance coaches. BMJ Open Sport Exerc Med. 2022;8. 10.1136/bmjsem-2021-001264. 10.1136/bmjsem-2021-001264 PMC8966548 35444812

[11] Ágh T, Kovács G, Supina D, Pawaskar M, Herman BK, Vokó Z, et al. A systematic review of the health-related quality of life and economic burdens of anorexia nervosa, bulimia nervosa, and binge eating disorder. Eat Weight Disord - Stud Anorex Bulim Obes. 2016;21:353–64. 10.1007/s40519-016-0264-x. 10.1007/s40519-016-0264-x PMC5010619 26942768

[12] Martinsen M, Sundgot-Borgen J. Higher Prevalence of Eating Disorders among Adolescent Elite Athletes than Controls. Med Sci Sports Exerc. 2013;45:1188. 10.1249/MSS.0b013e318281a939. 23274604 10.1249/MSS.0b013e318281a939

[13] Reardon CL, Hainline B, Aron CM, Baron D, Baum AL, Bindra A, et al. Mental health in elite athletes: International Olympic Committee consensus statement (2019). Br J Sports Med. 2019;53:667–99. 10.1136/bjsports-2019-100715. 31097450 10.1136/bjsports-2019-100715

[14] World Health Organization. International Classification of Diseases, Eleventh Revision (ICD-11). WHO; 2021.

[15] American Psychiatric Association. Diagnostisches und Statistisches Manual Psychischer Störungen DSM-5^®^. 2., korrigierte Auflage. Göttingen: Hogrefe; 2015.

[16] Esser-Seraphin AJ, Geiger S, Paslakis G, Muehlbauer T, Gradl-Dietsch G, Seitz J, et al. Übersichtsbeiträge (Reviews). Is disordered eating part of the game? A theoretical model in elite sports / Gehört gestörtes Essverhalten dazu? Ein theoretisches Modell im Leistungssport. Z Psychosom Med Psychother. 2025;71:189–99. 10.13109/zptm.2025.71.2.189. 40577561 10.13109/zptm.2025.71.2.189

[17] Plateau CR, Arcelus J, Leung N, Meyer C. Female athlete experiences of seeking and receiving treatment for an eating disorder. Eat Disord. 2017;25:273–7. 10.1080/10640266.2016.1269551. 28051927 10.1080/10640266.2016.1269551

[18] Fewell LK, Nickols R, Tierney AS, Levinson CA. Eating Disorders in Sport: Comparing Eating Disorder Symptomatology in Athletes and Non-Athletes During Intensive Eating Disorder Treatment. 2018. 10.1123/jcsp.2018-0046

[19] Halbeisen G, Laskowski N, Brandt G, Waschescio U, Paslakis G. Eating Disorders in Men. Dtsch Ärztebl Int. 2024;121:86–91. 10.3238/arztebl.m2023.0246. 38019152 10.3238/arztebl.m2023.0246 PMC11002438

[20] Hazzard VM, Schaefer LM, Mankowski A, Carson TL, Lipson SM, Fendrick C, et al. Development and validation of the Eating Disorders Screen for Athletes (EDSA): A brief screening tool for male and female athletes. Psychol Sport Exerc. 2020;50:101745. 10.1016/j.psychsport.2020.101745. 32733166 10.1016/j.psychsport.2020.101745 PMC7392177

[21] Burke LM, Lundy B, Fahrenholtz IL, Melin AK. Pitfalls of Conducting and Interpreting Estimates of Energy Availability in Free-Living Athletes. Int J Sport Nutr Exerc Metab. 2018;28:350–63. 10.1123/ijsnem.2018-0142. 30029584 10.1123/ijsnem.2018-0142

[22] de Borja C, Holtzman B, McCall LM, Carson TL, Moretti LJ, Farnsworth N, et al. Specific dietary practices in female athletes and their association with positive screening for disordered eating. J Eat Disord. 2021;9:50. 10.1186/s40337-021-00407-7. 33865448 10.1186/s40337-021-00407-7 PMC8052728

[23] Kontele I, Vassilakou T. Nutritional Risks among Adolescent Athletes with Disordered Eating. Children. 2021;8:715. 10.3390/children8080715. 34438606 10.3390/children8080715 PMC8394476

[24] Logue DM, Mahony L, Corish CA, Tobin D, Doherty R, O’Higgins G, et al. Athletes’ and Coaches’ Perceptions of Nutritional Advice: Eating More Food for Health and Performance. Nutrients. 2021;13:1925. 10.3390/nu13061925. 34205107 10.3390/nu13061925 PMC8227796

[25] Krentz EM, Warschburger P. A longitudinal investigation of sports-related risk factors for disordered eating in aesthetic sports. Scand J Med Sci Sports. 2013;23:303–10. 10.1111/j.1600-0838.2011.01380.x. 22093018 10.1111/j.1600-0838.2011.01380.x

[26] Lichtenstein MB, Johansen KK, Runge E, Hansen MB, Holmberg TT, Tarp K. Behind the athletic body: a clinical interview study of identification of eating disorder symptoms and diagnoses in elite athletes. BMJ Open Sport Exerc Med. 2022;8. 10.1136/bmjsem-2021-001265. 10.1136/bmjsem-2021-001265 PMC9214368 35813128

[27] Martinsen M, Bratland-Sanda S, Eriksson AK, Sundgot-Borgen J. Dieting to win or to be thin? A study of dieting and disordered eating among adolescent elite athletes and non-athlete controls. Br J Sports Med. 2010;44:70. 10.1136/bjsm.2009.068668. 20026698 10.1136/bjsm.2009.068668

[28] Uriegas NA, Winkelmann ZK, Pritchett K, Torres-McGehee TM. Examining Eating Attitudes and Behaviors in Collegiate Athletes, the Association Between Orthorexia Nervosa and Eating Disorders. Front Nutr. 2021;8. 10.3389/fnut.2021.763838. 10.3389/fnut.2021.763838 PMC8632486 34859033

[29] Piacentino D, Kotzalidis GD, del Casale A, Aromatario MR, Pomara C, Girardi P, et al. Anabolic-androgenic Steroid use and Psychopathology in Athletes. A Systematic Review. Curr Neuropharmacol. 2015;13:101–21. 10.2174/1570159X13666141210222725. 26074746 10.2174/1570159X13666141210222725 PMC4462035

[30] Abrahamsen FE, Roberts GC, Pensgaard AM. Achievement goals and gender effects on multidimensional anxiety in national elite sport. Psychol Sport Exerc. 2008;9:449–64. 10.1016/j.psychsport.2007.06.005.

[31] Schaal K, VanLoan MD, Hausswirth C, Casazza GA. Decreased energy availability during training overload is associated with non-functional overreaching and suppressed ovarian function in female runners. Appl Physiol Nutr Metab. 2021;46:1179–88. 10.1139/apnm-2020-0880. 33651630 10.1139/apnm-2020-0880

[32] Stellingwerff T, Heikura IA, Meeusen R, Bermon S, Seiler S, Mountjoy ML, et al. Overtraining Syndrome (OTS) and Relative Energy Deficiency in Sport (RED-S): Shared Pathways, Symptoms and Complexities. Sports Med. 2021;51:2251–80. 10.1007/s40279-021-01491-0. 34181189 10.1007/s40279-021-01491-0

[33] Budgett R. Overtraining syndrome. Br J Sports Med. 1990;24:231–6. 10.1136/bjsm.24.4.231. 2097018 10.1136/bjsm.24.4.231 PMC1478908

[34] Dipla K, Kraemer RR, Constantini NW, Hackney AC. Relative energy deficiency in sports (RED-S): elucidation of endocrine changes affecting the health of males and females. Hormones. 2021;20:35–47. 10.1007/s42000-020-00214-w. 32557402 10.1007/s42000-020-00214-w

[35] Cadegiani FA, Kater CE. Inter-correlations Among Clinical, Metabolic, and Biochemical Parameters and Their Predictive Value in Healthy and Overtrained Male Athletes: The EROS-CORRELATIONS Study. Front Endocrinol. 2019;10. 10.3389/fendo.2019.00858. 10.3389/fendo.2019.00858 PMC6914842 31920971

[36] Cadegiani FA, da Silva PHL, Abrao TCP, Kater CE. Diagnosis of Overtraining Syndrome: Results of the Endocrine and Metabolic Responses on Overtraining Syndrome Study: EROS-DIAGNOSIS. J Sports Med. 2020;2020:3937819. 10.1155/2020/3937819. 10.1155/2020/3937819 PMC7193300 32373644

[37] Carrard J, Rigort A-C, Appenzeller-Herzog C, Colledge F, Königstein K, Hinrichs T, et al. Diagnosing Overtraining Syndrome: A Scoping Review. Sports Health. 2022;14:665–73. 10.1177/19417381211044739. 34496702 10.1177/19417381211044739 PMC9460078

[38] Cadegiani FA. Special Topics on Overtraining Syndrome (OTS)/Paradoxical Deconditioning Syndrome (PDS). In: Cadegiani FA, editor. Overtraining Syndrome in Athletes: A Comprehensive Review and Novel Perspectives. Cham: Springer International Publishing; 2020. pp. 177–87. 10.1007/978-3-030-52628-3_10.

[39] Gould RJ, Ridout AJ, Newton JL. Relative Energy Deficiency in Sport (RED-S) in Adolescents – A Practical Review. Int J Sports Med. 2023;44:236–46. 10.1055/a-1947-3174. 36122585 10.1055/a-1947-3174

[40] Martinsen M, Holme I, Pensgaard AM, Torstveit MK, Sundgot-Borgen J. The Development of the Brief Eating Disorder in Athletes Questionnaire. Med Sci Sports Exerc. 2014;46:1666. 10.1249/MSS.0000000000000276. 24504432 10.1249/MSS.0000000000000276

[41] Equator Network. Reporting Guidelines. Equator Network. n.d. https://www.equator-network.org/reporting-guidelines/. Accessed 21 Oct 2024.

[42] Korevaar DA, Cohen JF, Reitsma JB, Bruns DE, Gatsonis CA, Glasziou PP, et al. Updating standards for reporting diagnostic accuracy: the development of STARD 2015. Res Integr Peer Rev. 2016;1:7. 10.1186/s41073-016-0014-7. 29451535 10.1186/s41073-016-0014-7 PMC5803584

[43] Lämmle L. Theoretische Konzeption, Diagnostik und Bedeutung von Motivation und Selbstregulation im (Hoch-)Leistungssport. Motivation, Selbstregulation und Leistungsexzellenz. Münster: LIT; 2011. pp. 91–112.

[44] Pfaff E. Zum Umstieg vom Sport in den Beruf. Sportpsychologie. 2004;34(2004)6, S. 4–11:4–11.

[45] Pope HG, Gruber AJ, Choi P, Olivardia R, Phillips KA. Muscle Dysmorphia: An Underrecognized Form of Body Dysmorphic Disorder. Psychosomatics. 1997;38:548–57. 10.1016/S0033-3182(97)71400-2. 9427852 10.1016/S0033-3182(97)71400-2

[46] Lundy B, Torstveit MK, Stenqvist TB, Burke LM, Garthe I, Slater GJ, et al. Screening for Low Energy Availability in Male Athletes: Attempted Validation of LEAM-Q. Nutrients. 2022;14:1873. 10.3390/nu14091873. 35565840 10.3390/nu14091873 PMC9101736

[47] Melin A, Tornberg ÅB, Skouby S, Faber J, Ritz C, Sjödin A, et al. The LEAF questionnaire: a screening tool for the identification of female athletes at risk for the female athlete triad. Br J Sports Med. 2014;48:540–5. 10.1136/bjsports-2013-093240. 24563388 10.1136/bjsports-2013-093240

[48] Milos G, Spindler A, Schnyder U, Fairburn CG. Instability of eating disorder diagnoses: prospective study. Br J Psychiatry. 2005;187:573–8. 10.1192/bjp.187.6.573. 16319411 10.1192/bjp.187.6.573 PMC2710504

[49] Garner DM, Garfinkel PE. The Eating Attitudes Test: an index of the symptoms of anorexia nervosa. Psychol Med. 1979;9:273–9. 10.1017/S0033291700030762. 472072 10.1017/s0033291700030762

[50] Haase AM. Weight perception in female athletes: Associations with disordered eating correlates and behavior. Eat Behav. 2011;12:64–7. 10.1016/j.eatbeh.2010.09.004. 21184976 10.1016/j.eatbeh.2010.09.004

[51] Kennedy SF, Kovan J, Werner E, Mancine R, Gusfa D, Kleiman H. Initial validation of a screening tool for disordered eating in adolescent athletes. J Eat Disord. 2021;9:21. 10.1186/s40337-020-00364-7. 33588900 10.1186/s40337-020-00364-7 PMC7885388

[52] Fairburn CG, Cooper Z, O’Connor M. Eating Disorder Examination (Edition 16.0D). Cognitive Behavior Therapy and Eating Disorders. New York, NY: Guilford Press; 2008. pp. 265–308.

[53] Garner DM. Eating Disorder Inventory-2: Professional Manual. Psychological Assessment Resources; 1991.

[54] Chappell AJ, Simper TN. Nutritional Peak Week and Competition Day Strategies of Competitive Natural Bodybuilders. Sports. 2018;6:126. 10.3390/sports6040126. 30352979 10.3390/sports6040126 PMC6315482

[55] Escalante G, Stevenson SW, Barakat C, Aragon AA, Schoenfeld BJ. Peak week recommendations for bodybuilders: an evidence based approach. BMC Sports Sci Med Rehabil. 2021;13:68. 10.1186/s13102-021-00296-y. 34120635 10.1186/s13102-021-00296-y PMC8201693

[56] Galli N, Petrie TA, Reel JJ, Chatterton JM, Baghurst TM. Assessing the validity of the Weight Pressures in Sport Scale for Male Athletes. Psychol Men Masculinity. 2014;15:170–80. 10.1037/a0031762.

[57] Reel JJ, SooHoo S, Petrie TA, Greenleaf C, Carter JE. Slimming Down for Sport: Developing a Weight Pressures in Sport Measure for Female Athletes. J Clin Sport Psychol. 2010;4:99–111. 10.1123/jcsp.4.2.99.

[58] Hildebrandt T, Langenbucher J, Schlundt DG. Muscularity concerns among men: development of attitudinal and perceptual measures. Body Image. 2004;1:169–81. 10.1016/j.bodyim.2004.01.001. 18089149 10.1016/j.bodyim.2004.01.001

[59] Compte EJ, Cattle CJ, Lavender JM, Murray SB, Brown TA, Capriotti MR, et al. Psychometric evaluation of the Muscle Dysmorphic Disorder Inventory (MDDI) among cisgender gay men and cisgender lesbian women. Body Image. 2021;38:241–50. 10.1016/j.bodyim.2021.04.008. 33962223 10.1016/j.bodyim.2021.04.008 PMC8635416

[60] Longobardi C, Prino LE, Fabris MA, Settanni M. Muscle dysmorphia and psychopathology: Findings from an Italian sample of male bodybuilders. Psychiatry Res. 2017;256:231–6. 10.1016/j.psychres.2017.06.065. 28646788 10.1016/j.psychres.2017.06.065

[61] Vardardottir B, Olafsdottir AS, Gudmundsdottir SL. Body dissatisfaction, disordered eating and exercise behaviours: associations with symptoms of REDs in male and female athletes. BMJ Open Sport Exerc Med. 2023;9. 10.1136/bmjsem-2023-001731. 10.1136/bmjsem-2023-001731 PMC10860738 38348179

[62] Garner DM. Eating Disorder Inventory-3: Professional Manual. Lutz, FL: Psychological Assessment Resources; 2004.

[63] Punzi C, Tieri P, Girelli L, Petti M. Network-based validation of the psychometric questionnaire EDI-3 for the assessment of eating disorders. Sci Rep. 2023;13:1578. 10.1038/s41598-023-28743-5. 36709357 10.1038/s41598-023-28743-5 PMC9884211

[64] Garner DM, Olmsted MP, Polivy J, Garfinkel PE. Comparison Between Weight-Preoccupied Women and Anorexia Nervosa. Biopsychosoc Sci Med. 1984;46:255. 10.1097/00006842-198405000-00007 6739685

[65] Chapa DAN, Hagan KE, Forbush KT, Perko VL, Sorokina DA, Alasmar AY, et al. The Athletes’ Relationships with Training scale (ART): A self-report measure of unhealthy training behaviors associated with eating disorders. Int J Eat Disord. 2018;51:1080–9. 10.1002/eat.22960. 30312490 10.1002/eat.22960 PMC6519369

[66] Borg G. Perceived exertion as an indicator of somatic stress. Scand J Rehabil Med. 1970;2:92–8. 5523831

[67] Borg G. Borg’s Perceived Exertion And Pain Scales. Human Kinetics; 1998.

[68] Borg G. Psychophysical bases of perceived exertion. Med Sci Sports Exerc. 1982;14:377. 7154893

[69] Dawes HN, Barker KL, Cockburn J, Roach N, Scott O, Wade D. Borg’s Rating of Perceived Exertion Scales: Do the Verbal Anchors Mean the Same for Different Clinical Groups? Arch Phys Med Rehabil. 2005;86:912–6. 10.1016/j.apmr.2004.10.043. 15895336 10.1016/j.apmr.2004.10.043

[70] Liu H, Yang W, Liu H, Bao D, Cui Y, Ho IMK, et al. A meta-analysis of the criterion-related validity of Session-RPE scales in adolescent athletes. BMC Sports Sci Med Rehabil. 2023;15:101. 10.1186/s13102-023-00712-5. 37573328 10.1186/s13102-023-00712-5 PMC10422765

[71] Ieno C, Baldassarre R, Pennacchi M, Torre AL, Bonifazi M, Piacentini MF. Monitoring Rating of Perceived Exertion Time in Zone: A Novel Method to Quantify Training Load in Elite Open-Water Swimmers? Int J Sports Physiol Perform. 2021;16:1551–5. 10.1123/ijspp.2020-0707. 33761462 10.1123/ijspp.2020-0707

[72] Micklewright D, St Clair Gibson A, Gladwell V, Al Salman A. Development and Validity of the Rating-of-Fatigue Scale. Sports Med. 2017;47:2375–93. 10.1007/s40279-017-0711-5. 28283993 10.1007/s40279-017-0711-5 PMC5633636

[73] Shacham S. A Shortened Version of the Profile of Mood States. J Pers Assess. 1983;47:305–6. 10.1207/s15327752jpa4703_14. 6886962 10.1207/s15327752jpa4703_14

[74] Grove B, Prapavessis H. Preliminary evidence for the reliability and validity of an abbreviated Profile of Mood States. Int J Sport Psychol. 1992;23:93–109.

[75] Trakman GL, Forsyth A, Hoye R, Belski R. Development and validation of a brief general and sports nutrition knowledge questionnaire and assessment of athletes’ nutrition knowledge. J Int Soc Sports Nutr. 2018;15:17. 10.1186/s12970-018-0223-1. 29713248 10.1186/s12970-018-0223-1 PMC5907737

[76] Trakman GL, Brown F, Forsyth A, Belski R. Modifications to the nutrition for sport knowledge questionnaire (NSQK) and abridged nutrition for sport knowledge questionnaire (ANSKQ). J Int Soc Sports Nutr. 2019;16:26. 10.1186/s12970-019-0293-8. 31253151 10.1186/s12970-019-0293-8 PMC6598343

[77] Brytek-Matera A, Obeid S, Donini LM, Rogoza M, Marchlewska M, Plichta M, et al. Psychometric properties of the ORTO-R in a community-based sample of women and men from Poland. J Eat Disord. 2023;11:9. 10.1186/s40337-023-00734-x. 36658619 10.1186/s40337-023-00734-x PMC9850681

[78] Donini LM, Marsili D, Graziani MP, Imbriale M, Cannella C. Orthorexia nervosa: Validation of a diagnosis questionnaire. Eat Weight Disord - Stud Anorex Bulim Obes. 2005;10:e28–32. 10.1007/BF03327537. 10.1007/BF03327537 16682853

[79] Rogoza R, Donini LM. Introducing ORTO-R: a revision of ORTO-15. Eat Weight Disord - Stud Anorex Bulim Obes. 2021;26:887–95. 10.1007/s40519-020-00924-5. 10.1007/s40519-020-00924-5 PMC8004519 32436165

[80] Rogoza R. Investigating the structure of ORTO-15: a meta-analytical simulation study. Eat Weight Disord - Stud Anorex Bulim Obes. 2019;24:363–5. 10.1007/s40519-018-0621-z. 10.1007/s40519-018-0621-z PMC6441397 30498988

[81] Özdengül F, Yargic MP, Solak R, Yaylali O, Kurklu GB. Assessment of orthorexia nervosa via ORTO-R scores of Turkish recreational and competitive athletes and sedentary individuals: a cross-sectional questionnaire study. Eat Weight Disord - Stud Anorex Bulim Obes. 2021;26:1111–8. 10.1007/s40519-020-01006-2. 10.1007/s40519-020-01006-2 32918258

[82] Rice SM, Parker AG, Mawren D, Clifton P, Harcourt P, Llyod M, et al. Preliminary psychometric validation of a brief screening tool for athlete mental health among male elite athletes: the Athlete Psychological Strain Questionnaire. Int J Sport Exerc Psychol. 2019;18:850–65. 10.1080/1612197X.2019.1611900.

[83] Rice SM, Olive L, Gouttebarge V, Parker AG, Clifton P, Harcourt P, et al. Mental health screening: severity and cut-off point sensitivity of the Athlete Psychological Strain Questionnaire in male and female elite athletes. BMJ Open Sport Exerc Med. 2020;6. 10.1136/bmjsem-2019-000712. 10.1136/bmjsem-2019-000712 PMC7101042 32231792

[84] Kroenke K, Spitzer RL, Williams JBW, Löwe B. An Ultra-Brief Screening Scale for Anxiety and Depression: The PHQ–4. Psychosomatics. 2009;50:613–21. 10.1016/S0033-3182(09)70864-3. 19996233 10.1176/appi.psy.50.6.613

[85] Löwe B, Wahl I, Rose M, Spitzer C, Glaesmer H, Wingenfeld K, et al. A 4-item measure of depression and anxiety: Validation and standardization of the Patient Health Questionnaire-4 (PHQ-4) in the general population. J Affect Disord. 2010;122:86–95. 10.1016/j.jad.2009.06.019. 19616305 10.1016/j.jad.2009.06.019

[86] World Health Organization. International Classification of Diseases, Eleventh Revision (ICD-11). 2019.

[87] World Health Organization. A healthy lifestyle - WHO recommendations. World Health Organization (WHO). 2010. https://www.who.int/europe/news-room/fact-sheets/item/a-healthy-lifestyle---who-recommendations. Accessed 9 July 2025.

[88] Thibault R, Genton L, Pichard C. Body composition: Why, when and for who? Clin Nutr. 2012;31:435–47. 10.1016/j.clnu.2011.12.011. 22296871 10.1016/j.clnu.2011.12.011

[89] Gerrior S, Juan W, Basiotis P. An Easy Approach to Calculating Estimated Energy Requirements. Prev Chronic Dis; 2006. PMC1784117 16978504

[90] Faul F, Erdfelder E, Buchner A, Lang A-G. Statistical power analyses using G*Power 3.1: Tests for correlation and regression analyses. Behav Res Methods. 2009;41:1149–60. 10.3758/BRM.41.4.1149. 19897823 10.3758/BRM.41.4.1149

[91] Jankauskiene R, Baceviciene M, Trinkuniene L. Examining Body Appreciation and Disordered Eating In Adolescents of Different Sports Practice: Cross-Sectional Study. Int J Environ Res Public Health. 2020;17:4044. 10.3390/ijerph17114044. 32517115 10.3390/ijerph17114044 PMC7312658

[92] Karrer Y, Halioua R, Mötteli S, Iff S, Seifritz E, Jäger M, et al. Disordered eating and eating disorders in male elite athletes: a scoping review. BMJ Open Sport Exerc Med. 2020;6. 10.1136/bmjsem-2020-000801. 10.1136/bmjsem-2020-000801 PMC7642204 33178441

[93] Rousselet M, Guérineau B, Paruit MC, Guinot M, Lise S, Destrube B, et al. Disordered eating in French high-level athletes: association with type of sport, doping behavior, and psychological features. Eat Weight Disord - Stud Anorex Bulim Obes. 2017;22:61–8. 10.1007/s40519-016-0342-0. 10.1007/s40519-016-0342-0 27838862

[94] Ghazzawi HA, Nimer LS, Haddad AJ, Alhaj OA, Amawi AT, Pandi-Perumal SR, et al. A systematic review, meta-analysis, and meta-regression of the prevalence of self-reported disordered eating and associated factors among athletes worldwide. J Eat Disord. 2024;12:24. 10.1186/s40337-024-00982-5. 38326925 10.1186/s40337-024-00982-5 PMC10851573

[95] Chen H, Cohen P, Chen S. How Big is a Big Odds Ratio? Interpreting the Magnitudes of Odds Ratios in Epidemiological Studies. Commun Stat - Simul Comput. 2010;39:860–4. 10.1080/03610911003650383.

[96] Cohen J. Statistical power analysis for the behavioral sciences. 2. ed., reprint. New York, NY: Lawrence Erlbaum Associates; 1988.

[97] Youden WJ. Index for rating diagnostic tests. Cancer. 1950;3:32–5. 10.1002/1097-0142(. 1950)3:1%3C32::aid-cncr2820030106%3E3.0.co;2-3. 15405679 10.1002/1097-0142(1950)3:1<32::aid-cncr2820030106>3.0.co;2-3

[98] European Union. General Data Protection Regulation. 2016.

[99] World Medical Association. WMA Declaration of Helsinki - Ethical Principles for Medical Research Involving Human Participants. 2024. 10.1001/jama.2024.21972 39425955

[100] Eichstadt M, Luzier J, Cho D, Weisenmuller C. Eating Disorders in Male Athletes. Sports Health. 2020;12:327–33. 10.1177/1941738120928991. 32525767 10.1177/1941738120928991 PMC7787561

[101] Holtzman B, Ackerman KE. Recommendations and Nutritional Considerations for Female Athletes: Health and Performance. Sports Med. 2021;51:43–57. 10.1007/s40279-021-01508-8. 34515972 10.1007/s40279-021-01508-8 PMC8566643

[102] Rosinska M, Soós D, Gálvez Solé L, Ibáñez-Caparrós A, Thiel A, Zipfel S, et al. Athletes with eating disorders: clinical-psychopathological features and gender differences. J Eat Disord. 2025;13:40. 10.1186/s40337-025-01221-1. 39994756 10.1186/s40337-025-01221-1 PMC11853795

[103] Araújo CGS, Scharhag J. Athlete: a working definition for medical and health sciences research. Scand J Med Sci Sports. 2016;26:4–7. 10.1111/sms.12632. 26750158 10.1111/sms.12632

[104] Skorski S, Hecksteden A. Coping With the Small Sample–Small Relevant Effects Dilemma in Elite Sport Research. Int J Sports Physiol Perform. 2021;16:1559–60. 10.1123/ijspp.2021-0467. 34653960 10.1123/ijspp.2021-0467

[105] Schweizer G, Furley P. Reproducible research in sport and exercise psychology: The role of sample sizes. Psychol Sport Exerc. 2016;23:114–22. 10.1016/j.psychsport.2015.11.005.

